# Flexible females: nutritional state influences biparental cooperation in a burying beetle

**DOI:** 10.1093/beheco/arae009

**Published:** 2024-02-24

**Authors:** Georgia A Lambert, Per T Smiseth

**Affiliations:** Institute of Ecology and Evolution, University of Edinburgh, Charlotte Auerbach Road, Edinburgh EH9 3FL, UK; Institute of Ecology and Evolution, University of Edinburgh, Charlotte Auerbach Road, Edinburgh EH9 3FL, UK

**Keywords:** cooperation, nutritional state, *Nicrophorus vespilloides*, parental care, sexual conflict

## Abstract

In species that provide biparental care, there is a sexual conflict between parents over how much each should contribute toward caring for their joint offspring. Theoretical models for the resolution of this conflict through behavioral negotiation between parents assume that parents cannot assess their partner’s state directly but do so indirectly by monitoring their partner’s contribution. Here, we test whether parents can assess their partner’s state directly by investigating the effect of nutritional state on cooperation between parents in the burying beetle *Nicrophorus vespilloides*. We used a two-by-two factorial design, in which a well-fed or food-deprived female was paired with a well-fed or food-deprived male. We found that females adjusted their level of care in response to both their own nutritional state and that of their partner and that these decisions were independent of their partner’s contribution. We found no evidence that males responded directly to the nutritional state. Males instead responded indirectly based on the contribution of their partner. Our results suggest that parents are able to assess the state of their partner, in contrast to what has been assumed, and that these assessments play an important role in the mediation of sexual conflict between caring parents.

## Teaser

We found that the amount of time female burying beetles spent caring for their offspring was dependent on their partner’s health as well as their own health. This contradicts an established assumption that parents do not make decisions about parental care directly based on their partner’s health. Our results suggest that females can assess the health status of their partners and that these assessments play an important role in parental cooperation.

## Introduction

There is sexual conflict between parents who provide biparental care since the benefits of care depend on the combined effort of the two parents, whereas the costs of future reproduction are paid individually (Houston et al. 2005; Lessells 2012). Parents are, therefore, under selection to shift as much of the workload as possible onto their partner (Parker 1985). Theoretical models have identified several mechanisms by which this conflict may be resolved: negotiation, matching, or a sealed-bid decision. Negotiation involves a parent responding to a decrease in the amount of care contributed by its partner by increasing its own contribution but only such that it incompletely compensates for the partner’s decrease (McNamara et al. 1999). Matching involves a parent responding to a change in its partner’s contribution by increasing when its partner increases or decreasing when its partner decreases its contribution (Johnstone and Hinde 2006). A sealed-bid decision is when a parent makes an initial decision about how much care to provide that is independent of its partner’s contribution (Houston and Davies 1985). There is empirical evidence supporting negotiation (Harrison et al. 2009; Pilakouta et al. 2015), matching (Hinde 2006; Lendvai et al. 2018), and sealed-bid decisions (Schwagmeyer et al. 2002; Mattey and Smiseth 2015; Pilakouta et al. 2015) across different taxa; however, in birds, the most studied taxonomic group, negotiation is thought to be the predominant mechanism for cooperation (Harrison et al. 2009).

Variations in the state of the parents may play an important role in determining how sexual conflict over care is resolved. Here, we refer to “state” as any attribute of an individual, such as its nutritional status, body size, inbreeding status, infection status, or age, which is likely to affect its contribution toward care and/or its partner’s contribution. A focal parent is likely to adjust its contribution based on its own state since variation in state may influence the cost and/or benefit functions associated with providing care (Smiseth 2017). The focal parent may also adjust its contribution based on its partner’s state since this may provide information about the likely future contribution of its partner and/or the potential value of the joint brood. Theoretical models for negotiation as a mechanism of conflict resolution assume that a focal parent cannot assess its partner’s state directly and that it does so indirectly by monitoring its contribution (McNamara et al. 1999; McNamara and Wolf 2022). These models emphasize that variation in the state of parents plays a key role in promoting the evolution of negotiation as a behavioral mechanism of conflict resolution when the focal parent cannot assess its partner’s state directly. Meanwhile, more recent research has shown that a focal parent can respond directly to variation in multiple states of its partner and that it also independently responds to its partner’s contribution (Mattey and Smiseth 2015; Pilakouta et al. 2015). Thus, there is a need for more work exploring the role of state in negotiation in particular, and the mediation of sexual conflict between parents in general.

Here, we investigate the effects of a temporary and reversible state, that is, nutritional state, on the dynamics of biparental cooperation. Prior studies investigating the effects of state on biparental cooperation have focused on permanent states, such as inbreeding status and adult body size, which remain constant throughout adulthood. For a permanent state, we expect a focal parent to adjust its contribution to its own state and that of its partner since state affects the ability to provide care. In support of this, previous studies have found that individuals adjust the amount of care they contribute based on their own body size and that of their partner (Pilakouta et al. 2015) and the inbreeding status of their partner (Mattey and Smiseth 2015). In contrast, we might expect different dynamics for a temporary and reversible state, such as being malnourished or infected with a pathogen, given that a focal individual may improve its state by reducing its own contribution to care. Thus, for temporary and reversible states, biparental care may facilitate recovery by allowing a malnourished or infected individual to invest more in its own recovery, thereby shifting a greater amount of the workload over to its partner.

We tested this idea by manipulating the nutritional state of females and males of the burying beetle *Nicrophorus vespilloides* and measuring the effect on how a focal parent responded to its own state and that of its partner. Beetles in the genus *Nicrophorus* are well suited to test this idea. Firstly, parents provide biparental care, and the level of care parents provide is flexible. *Nicrophorus vespilloides* breeds on a small vertebrate carcass that serves as a joint food source for both parents and their offspring. Female and male parents provide care for their offspring, including preparing and maintaining the carcass by spreading antimicrobials onto it, provisioning pre-digested carrion to their offspring, and guarding their offspring against conspecific intruders (Eggert et al. 1998; Scott 1998). Although both parents are capable of all activities, females tend to spend more time food provisioning to the larvae (Eggert et al. 1998; Smiseth and Moore 2002; Smiseth et al. 2005), while males spend more time maintaining the carcass (Smiseth et al. 2005). Secondly, it is relatively straightforward to manipulate an individual’s nutritional state by simply subjecting them to food deprivation for 7 days before breeding. Prior work also shows that food-deprived parents feed more during breeding than well-fed parents (Keppner et al. 2018) and that females respond to their own nutritional state since food-deprived females spend less time maintaining the carcass and provisioning food to their larvae in comparison to well-fed females (Richardson and Smiseth 2019a). However, there is no information on the effects of nutritional state on the dynamics of biparental cooperation.

Our aim was to test whether parents adjust the level of care they provide based on the nutritional state of their partner and whether this was conditional upon their own nutritional state. To meet this aim, we used a two-by-two factorial design where a well-fed or food-deprived female was paired with a well-fed or food-deprived male. We predict that a food-deprived focal parent will decrease the level of care it provides when paired with a well-fed partner since its partner is able to compensate for a reduction in care due to the poor state of the focal parent. In contrast, a food-deprived focal parent may not be able to decrease the level of care it provides when paired with a food-deprived partner since its partner is less able to compensate. We also predict that the partner of a food-deprived parent will incompletely compensate for the expected lower level of care provided by the food-deprived parent and that food-deprived individuals would provide less care than well-fed individuals since food-deprived individuals are likely to pay a higher cost of providing care (Richardson and Smiseth 2019a). To determine whether a focal parent assessed its partner’s state directly or indirectly by monitoring its partner’s contribution, we added the partner’s contribution to the model to test whether it accounted for any observed effects of the partner’s state on the focal parent. We tested if food-deprived individuals prioritize improving their own nutritional state when compared to well-fed parents as a means to recover from any potential costs of food deprivation to their ability to invest in future reproduction. We predict that food-deprived individuals would consume more carrion and thus gain more mass during breeding than well-fed individuals. Finally, we tested for the effects of nutritional state on the size and quality of the joint brood. We expect that the broods of food-deprived parents will be smaller and of worse quality since we expect food-deprived parents to provide less care and to consume more carrion (a joint food source), which would reduce the amount of food available for the larvae.

## METHODS

### General methodology

We used beetles from an outbred laboratory population originally collected in Edinburgh, UK and maintained at the University of Edinburgh. We housed all adults in the stock population individually in clear plastic containers (12 cm × 8 cm × 2 cm) lined with moist soil and fed them raw organic beef twice a week. The stock population was kept at 20 °C under a 16:8 h light:dark cycle.

### Experimental design

We used a two-by-two factorial design with the following treatments: a food-deprived female paired with a food-deprived male (*n* = 30), a food-deprived female paired with a well-fed male (*n* = 30), a well-fed female paired with a food-deprived male (*n* = 32), and a well-fed female paired with a well-fed male (*n* = 31). All individuals used in the experiment were at least 10 days post-eclosion to ensure they had reached sexual maturity and that feeding treatment had no effect on the rate of maturation. We weighed all individuals before assigning them to one of the four treatments in our experiment. We used established protocols to produce well-fed and food-deprived females and males. Food-deprived individuals were not fed during the 7-day treatment, whereas well-fed individuals were fed twice with organic beef (approximately 0.3 g) during this period. This level of food deprivation was chosen since it leads to a significant drop in weight without causing an increase in mortality (Richardson and Smiseth 2019a, 2019b). After 7 days, we weighed all individuals to measure their post-treatment and pre-breeding mass. We used this to calculate weight change during the treatment period and confirm that our food deprivation treatment had the intended effect on the nutritional state (see Results).

Immediately after weighing, we paired up males and females at random, taking care to avoid mating between close relatives. We transferred each pair into a clear plastic container (17 cm × 12 cm × 6 cm) lined with 1 cm of moist soil. Each pair was provided with a freshly thawed mouse carcass (Livefoods Direct Ltd) of a standardized size (15–20 g; M ± SE = 18.47 ± 0.11 g) to initiate breeding. After 48 h, when the eggs had been laid but before the larvae had begun hatching, we moved the female, the male, and their carcass into a new clear plastic container (17 cm × 12 cm × 6 cm) lined with fresh moist soil. We allocated each pair a foster brood consisting of 20 newly hatched larvae from at least two different mothers. We chose this brood size since it is close to the average brood size for *N. vespilloides* (21 larvae; Smiseth and Moore 2002). We used a standardized brood size to control for potential confounding effects due to variation in brood size. Such confounding effects might arise because our treatment might affect the number of eggs laid (Steiger et al. 2007) and because brood size is known to affect the amount of care provided by parents (Smiseth and Moore 2002). We allocated a foster brood to a pair only after their eggs had started hatching since parents use temporal kin recognition and so would kill larvae that arrive at the carcass before their own eggs started hatching (Müller and Eggert 1990).

We conducted behavioral observations 24 h (± 15 min) after we allocated pairs a foster brood since this is when parents provide the highest level of care in this species (Smiseth et al. 2003). We did the observations under red light using instantaneous sampling of female and male behavior every minute for 30 min consistent with established protocols (Smiseth and Moore 2002). At each scan, we recorded whether females and males were providing direct care, indirect care, or consuming carrion. We used the number of scans as a proxy for the amount of time an individual spent providing care or consuming carrion. Direct care included provisioning food to the larvae (mouth-to-mouth contact between the parent and at least one larvae) and grooming the larvae. Indirect care included maintaining the carcass (spreading antimicrobial secretions onto its surface or modifying the position of the carcass) and guarding the brood and carcass against competitors or predators (standing still on the carcass facing away from the brood). After the observations, we left the pairs to care for their brood until the larvae dispersed from the carcass approximately 5 days later, upon which we recorded average larval mass and the proportion of larvae that survived to dispersal as measures of offspring performance, and female and male post-breeding mass to allow us to calculate individual mass change during breeding.

### Statistical analysis

All statistical analyses were conducted using R version 3.6.1 (R Core Team 2021) with the packages car (Fox and Weisberg 2019), MASS (Venables and Ripley 2022), and glmmTMB (Brooks et al. 2017). We used zero-inflated binomial models in our analyses on the amount of time spent providing direct care by females and males since the data for this behavior showed minor zero inflation. We used binomial models in our analysis on time spent providing indirect care and consuming carrion by females and males and larval survival to dispersal. In all these models, we included observation level as a random effect to account for over-dispersion (Harrison 2015). We used linear models for data on female and male mass change during the food deprivation treatment, female and male mass change during breeding, and mean larval mass at dispersal.

To determine whether the focal parent responded directly to the nutritional state of its partner or indirectly to its partner’s contribution, we compared models where we included and excluded the amount of time spent providing direct or indirect care or consuming carrion by the partner as a factor (Mattey and Smiseth 2015; Pilakouta et al. 2015). If including this factor reduced or removed any effect of the partner’s nutritional state on the amount of time spent providing direct or indirect care or consuming carrion by the focal parent, we interpreted this as evidence that the focal parent responded indirectly to the contribution of its partner, as expected by theoretical models of negotiation. However, if including this factor did not negate the effect of the partner’s nutritional state on the amount of time spent providing direct or indirect care or consuming carrion by the focal parent, we interpreted this as evidence that the focal parent responded directly to the nutritional state of its partner. We note that our data on the responses of the focal parent to its partner’s contribution are correlational and that we therefore cannot demonstrate a causal relationship between the contributions of females and males to parental care.

## RESULTS

### Effects of food deprivation on male and female mass change

Food-deprived females and males lost more mass during the treatment period than well-fed females and males (females: estimate = −0.052 ± 0.005 g, *t* = −11.11, *P *< 0.001, males: estimate = −0.035 ± 0.004 g, *t* = −7.96, *P *< 0.001). This confirms that the food deprivation treatment had the intended effect of altering an individual’s nutritional state. There was no difference between female and male mass change during the food deprivation treatment (estimate = 0.009 ± 0.004 g, *t* = 1.63, *P* = 0.105).

### Effects of nutritional state on parental care and cooperation

**Figure 1:**
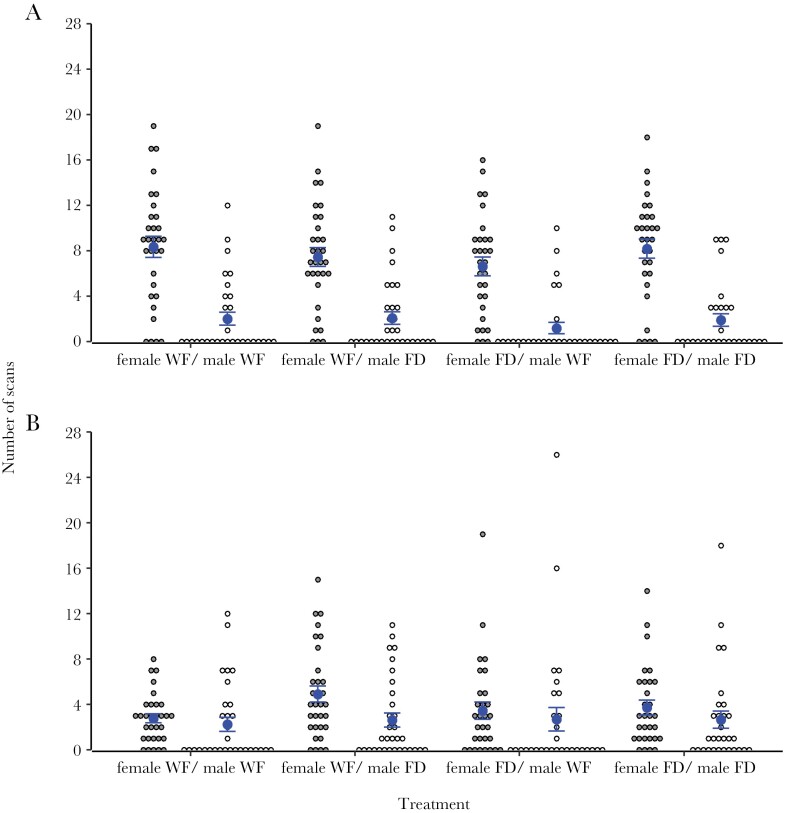
Comparison of the number of scans (out of 30) spent providing direct care (A) and indirect care (B) by well-fed (WF) or food-deprived (FD) females (gray points) and well-fed or food-deprived males (white points) caring for larvae during a 30-min behavioral observation (mean ± SE).

There was a significant effect of the interaction between a female’s nutritional state and the nutritional state of its partner on the amount of time spent providing direct care (Table 1). The estimate of this interaction effect was positive (Table 1), indicating that food-deprived females responded to being paired with a food-deprived male, rather than well-fed male, by more strongly increasing the amount of time they spent providing direct care than did well-fed females. Indeed, visual inspection of Figure 1 shows that food-deprived females spent more time providing direct care when paired with a food-derived male than when paired with a well-fed male, while well-fed females provided similar levels of care regardless of whether they were paired with food-deprived or well-fed males. Thus, females responded to the nutritional state of their partner, but any such response was conditional upon the female’s own nutritional state. There was also a significant main effect of female nutritional state on the amount of time spent providing direct care by females (Table 1, Figure 1). This finding is due to the interaction described above. There was no significant effect of the interaction between a male’s nutritional state and the nutritional state of its partner on the amount of time spent providing direct care (Table 1, Figure 1). There was no main effect of the partner’s nutritional state on the amount of time females and males spent providing direct care (Table 1, Figure 1). There was also no main effect of male nutritional state on the amount of time spent providing direct care by males (Table 1, Figure 1).

**Table 1:** Summary of statistical tests for the effects of nutritional state on bi-parental cooperation over providing direct and indirect care excluding and including (*) partner’s contribution in the model. The reference category for the focal parent’s nutritional state and the partner’s nutritional state was “well-fed.” Statistically significant *P* values (<0.05) are shown in bold.

Behavior	Focal parent’s nutritional state				Partner’s nutritional state				Interaction				Partner’s contribution			
	Est	SE	*z*	*P*	Est	SE	*z*	*P*	Est	SE	*z*	*P*	Est	SE	*z*	*P*
Female direct	−0.41	0.19	−2.11	**0.035**	−0.24	0.19	−1.26	0.208	0.63	0.27	2.33	**0.020**				
Female direct*	−0.43	0.20	−2.12	**0.034**	−0.19	0.20	−0.98	0.325	0.60	0.28	2.13	**0.033**	−0.08	0.03	−2.42	**0.016**
Female indirect	0.11	0.31	0.35	0.724	0.65	0.29	2.22	**0.027**	−0.47	0.42	−1.12	0.262				
Female indirect*	0.11	0.31	0.35	0.723	0.65	0.29	2.22	**0.027**	−0.47	0.42	−1.12	0.262	−0.00	0.03	−0.10	0.917
Male direct	−0.23	0.34	−0.67	0.503	0.15	0.47	0.31	0.753	−0.07	0.58	−0.12	0.908				
Male direct*	−0.17	0.33	−0.54	0.593	0.10	0.46	0.22	0.827	−0.06	0.57	−0.11	0.912	−0.07	0.03	−2.38	**0.017**
Male indirect	0.44	0.66	0.66	0.507	−0.12	0.69	−0.18	0.859	0.16	0.94	0.17	0.865				
Male indirect*	0.45	0.67	0.68	0.498	−0.12	0.69	−0.17	0.865	0.15	0.95	0.16	0.876	−0.01	0.07	−0.14	0.887

There was no effect of the interaction between a female’s nutritional state and the nutritional state of its partner on the amount of time females spent providing indirect care; however, females provided more indirect care when paired with a food-deprived male (Table 1, Figure 1). Thus, females altered the amount of indirect care they provided in response to their partner’s nutritional state but did so regardless of their own nutritional state. There was no significant effect of the interaction between a male’s nutritional state and the nutritional state of its partner on the amount of time spent providing direct care (Table 1, Figure 1). There was no main effect of the partner’s nutritional state on the amount of time males spent providing indirect care (Table 1, Figure 1). There was also no main effect of focal parent nutritional state on the amount of time spent providing indirect care by females or males (Table 1, Figure 1).

The partner’s contribution was also a predictor of female care, suggesting that females responded to the contribution of their partner. Females spent less time providing direct care as the amount of time males spent providing direct care increased (Table 1). Including or excluding the partner’s contribution did not alter the effect of the interaction between the focal parent’s nutritional state and the nutritional state of the partner on the amount of direct care provided by females (Table 1). This suggests that females responded independently to the state and contribution of their partner. Partner contribution was a predictor of male care, suggesting that males also responded to the contribution of their partner. Males spent less time providing direct care as the amount of time females spent providing direct care increased (Table 1). There was no relationship between the partner’s contribution and the amount of time spent providing indirect care by females or males (Table 1).

### Male and female mass change during breeding and time spent consuming carrion

**Figure 2:**
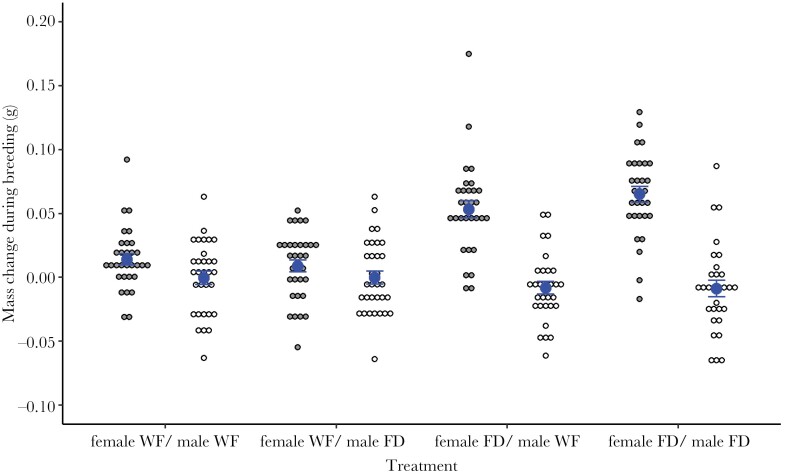
Comparison of the mass change during breeding by well-fed (WF) or food-deprived (FD) females (gray points) and well-fed or food-deprived males (white points) (mean ± SE).

In contrast to our predictions, there was no effect of the focal parent’s nutritional state, the partner’s nutritional state or the interaction between the two on the amount of time spent consuming carrion by females or males (Table 2). However, in line with our predictions, the female nutritional state affected female mass change during breeding with food-deprived females gaining more mass than well-fed females (Table 2, Figure 2). There was no effect of the male nutritional state on male mass change during breeding (Table 2). There was no effect of the interaction between the focal parent’s nutritional state and the partner’s nutritional state on the mass change of females or males during breeding (Table 2). There was also no effect of the partner’s nutritional state on the mass change of females or males during breeding (Table 2).

**Table 2:** Summary of statistical tests for the effects of nutritional state on bi-parental cooperation over consumption excluding and including (*) partner’s contribution in the model and mass change during breeding. The reference category for the focal parent’s nutritional state and the partner’s nutritional state was “well-fed.” Statistically significant *P* values (<0.05) are shown in bold.

Behavior	Focal parent’s nutritional state				Partner’s nutritional state				Interaction				Partner’s contribution			
	Est	SE	Test statistic	*P*	Est	SE	Test statistic	*P*	Est	SE	Test statistic	*P*	Est	SE	*z*	*P*
Female consumption	0.22	0.20	*z* = 1.10	0.273	−0.07	0.20	*z* = −0.36	0.716	−0.02	0.28	*z* = −0.05	0.957				
Female consumption*	0.22	0.20	*z* = 1.08	0.280	−0.09	0.20	*z* = -0.43	0.668	-0.00	0.28	*z* = -0.01	0.989	0.00	0.01	0.34	0.733
Female mass change	0.04	0.01	*t* = 5.13	**< 0.001**	−0.01	0.01	*t* = −0.66	0.509	0.02	0.01	*t* = 1.54	0.125				
Male consumption	0.78	1.28	*z* = 0.61	0.541	−0.61	1.22	*z* = −0.50	0.616	0.67	1.81	*z* = 0.37	0.713				
Male consumption*	0.83	1.28	*z* = 0.65	0.514	−0.54	1.22	*z* = −0.44	0.657	0.64	1.79	*z* = 0.36	0.720	−0.08	0.08	−1.02	0.309
Male mass change	0.00	0.01	*t* = 0.11	0.915	−0.01	0.01	*t* = −1.48	0.141	−0.00	0.01	*t* = −0.25	0.802				

### Offspring performance

Finally, we tested for the effects of the parents’ nutritional state on offspring performance upon dispersal. Average larval mass at dispersal was higher in broods cared for by food-deprived females (estimate = 0.019 ± 0.007 g, *t* = 2.73, *P* = 0.007). There was no effect of male nutritional state (estimate = 0.006 ± 0.007 g, *t* = 0.86, *P *= 0.392), and no effect of the interaction between female and male nutritional state (estimate = −0.016 ± 0.010 g, *t* = −1.668, *P *= 0.098) on average larval mass at dispersal. The proportion of larvae surviving to dispersal was not dependent on female nutritional state (estimate = 0.060 ± 0.236 g, *z* = 0. 25, *P *= 0.801), male nutritional state (estimate = 0.425 ± 0.234 g, *z* = 1.82, *P *= 0.069), or the interaction between the two (estimate = −0.159 ± 0.334 g, *z* = −0.48, *P *= 0.634).

## DISCUSSION

We found that females adjusted the amount of direct care they provided in response to the nutritional state of their partner and that this was conditional upon their own nutritional state. This interaction effect was likely driven by food-deprived females paired with well-fed males providing less care than food-deprived females paired with food-deprived males, while well-fed females provided similar levels of care regardless of whether they were paired with well-fed or food-deprived males. This finding is in line with our prediction that food-deprived individuals would reduce the amount of time they spend providing care if their partner is well-fed and is therefore capable of increasing its contribution. Reducing their contribution to care when paired with a well-fed male would enable food-deprived females to invest more into their own recovery whilst minimizing any detrimental effects to the joint brood. In support of this suggestion, food-deprived females gained more mass during breeding than well-fed females, which we will discuss in greater detail below. We also found that the amount of time females spent providing direct care was affected by the partner’s contribution. Females spent less time providing direct care when males spent more time providing direct care as predicted by negotiation models (McNamara et al. 1999). The inclusion of partner’s contribution in the model had little, if any, impact on the effect of the interaction or the partner’s state on the amount of time females spent providing direct care. This suggests that females responded directly to the nutritional state of their partner and that this response was independent of the response to the contribution of their partner.

Our results show that females adjust the amount of care they provide in response to the nutritional state of their partner and that they also independently respond to their partner’s contribution. This is interesting for several reasons. Firstly, our results add to growing evidence that responses to partner’s state and contribution are not mutually exclusive mechanisms for mediating conflict between caring parents (Mattey and Smiseth 2015; Pilakouta et al. 2015). A potential explanation for why parents respond to their partner’s state, as well as their partner’s contribution, is that state and contribution may provide somewhat different information about the partner’s expected future contribution to care (Pilakouta et al. 2015). Alternatively, parents may respond to their partner’s contribution to coordinate the distribution of parental care over time rather than to gain information about their partner’s expected contribution (Smiseth 2019). Thus, using two complementary mechanisms may enable parents to make a more accurate assessment of their partner’s expected contribution or the timing of their contribution and allow them to fine-tune their response. Secondly, our results are in contrast with theoretical models of negotiation, which assume that parents assess the state of their partner indirectly based on their partner’s contribution (McNamara et al. 1999). Currently, experiments focus on manipulating the contribution of a focal parent, typically via handicapping or mate removal, and then measuring any responses by its partner (Harrison et al. 2009). These designs may produce evidence that is biased toward negotiation since they negate the opportunity to test whether parents directly assess and respond to the state of their partner. As such, we suggest that future experiments are designed to reflect that multiple mechanisms may be involved in resolving sexual conflict, including direct responses to state as shown here. In a laboratory environment, this could be achieved by manipulating and measuring the effect of state, in addition to partner contribution, on parental behavior. In situations where manipulating state is impractical or unethical, including observational data on parental state in addition to data on partner contribution may also provide further insight. In sum, our findings highlight the need for more work exploring how multiple mechanisms may play a role in the dynamic balance between cooperation and conflict in species with biparental care.

We found a different pattern for indirect care by females in comparison to the results for direct care by females as discussed above. Females spent more time providing indirect care when paired with a food-deprived partner, regardless of their own nutritional state and their partner’s contribution. This result confirms that females adjust their contribution based on the state of their partner, although, in this case, this response was independent of their own state. The absence of an interaction effect between the focal parent nutritional state and partner nutritional state is surprising given that we expected individuals to respond to their partner being food-deprived only if they were well-fed and, therefore, capable of increasing their contribution. We found that both food-deprived and well-fed females increased the amount of time they spent providing indirect care when paired with a food-deprived male. A potential explanation is that providing indirect care is less energetically costly to females than providing direct care, and so they were able to increase the amount of indirect care they provide in response to male state even when food-deprived. Multiple studies have explored the cost of increased brood size (a strong predictor of the amount of direct care females provide) (Ward et al. 2009; Ratz and Smiseth 2018), but there is no information on the cost of providing indirect care alone or a direct comparison between direct and indirect care. To explore this idea, future work could test the energetic cost of providing direct and indirect care, potentially by simultaneously manipulating brood size and carcass size and measuring the effect on females.

Our results imply that females can assess the nutritional state of their partner and add to evidence that parents in *N. vespilloides* can assess and respond to various states of their partner, such as their inbreeding status and body size, independently of the partner’s contribution (Mattey and Smiseth 2015; Pilakouta et al. 2015). This raises questions as to how females do so. Our study was not designed to investigate the potential mechanisms that could be involved. Nevertheless, based on prior work, we suggest that cuticular chemicals are likely candidates. There is good evidence that cuticular chemicals play an important role in partner recognition in *N. vespilloides* (Steiger et al. 2007; Keppner et al. 2017), and there is also evidence that cuticular hydrocarbons are indicators of diet (Steiger et al. 2007; Fedina et al. 2012) and health (Beani et al. 2019) in *N. vespilloides* as well as other insects. Thus, it seems likely that malnutrition may alter an individual’s cuticular chemical profile, thereby allowing its partner to judge its nutritional state. We suggest future work further investigates the potential role of chemical cues as a mechanism mediating how parents are able to assess various cryptic states of their partner.

We found that males adjusted the amount of care they provided in response to the contribution of their partner, but there was no evidence that males responded to their partner’s nutritional state, their own nutritional state, or the interaction between the two. Instead, males decreased the time spent providing direct care as the amount of time females spent providing direct care increased, as predicted by negotiation models (McNamara et al. 1999). One explanation for this result is that males are unable to directly respond to the state of their partner and instead rely on the contribution of their partner as a measure of their ability to provide care. However, this seems unlikely given that females responded directly to the nutritional state of their partner, and that prior studies on *N. vespilloides* show that males respond to other components of their partner’s state (Mattey and Smiseth 2015; Pilakouta et al. 2015). Our results add to a growing list of sex differences in caring behavior in *N. vespilloides* (Smiseth and Moore 2004; Walling et al. 2008; Shippi et al. 2018). Currently, we have a poor understanding of what may be driving these differences, and this is an area that would warrant further work.

The main aim of this study was to investigate whether the effects of temporary states on the dynamics of biparental cooperation differ from those reported previously for permanent states (Mattey and Smiseth 2015; Pilakouta et al. 2015). We expected differences given that a parent has the opportunity to improve its state by reducing its contribution toward providing care for temporary and reversible states but not for permanent states. As such, we expected food-deprived individuals to provide less care than those in a comparably poor permanent state since, in addition to their reduced ability to provide care, they also have the incentive to invest in their own state whilst their partner incompletely compensates. There are both similarities and differences between the effects of nutritional state and previously tested permanent states. We found that food-deprived females spent less time providing direct care when paired with a well-fed male. Similarly, a previous study found that small females spend more time providing direct care when paired with a small male (Pilakouta et al. 2015). In both cases, females in a comparatively worse state only reduced the level of care they provided if their partner was in a better state and was able to compensate. In contrast, previous work found that inbred females spent the same amount of time providing direct care regardless of their partners’ state but that outbred females provided more care when paired with an inbred male (Mattey and Smiseth 2015). The challenge with making comparisons across different components of state is that this requires some way to calibrate the impact of states on the individual’s ability to provide care. For example, we cannot know whether our treatment of 7 days of food deprivation is equivalent to being of a particular size difference or a particular difference in inbreeding coefficients. As such, it is difficult to make true comparisons of the impact of nutritional state and body size or inbreeding state on parental cooperation. To overcome this, future studies may use a standardized test, such as measuring some aspect of performance, to calibrate the effect of different states on individuals. This would allow more accurate comparisons of any differential effects of temporary and permanent states on parental cooperation.

Parental mass change over the breeding attempt provided some insight into whether food-deprived individuals used the breeding attempt as an opportunity to recover from being in a temporarily worse state. We found no effect of the interaction between female and male nutritional state on female or male mass change during breeding to support this suggestion. However, food-deprived females gained more mass during breeding than well-fed females. In contrast, Keppner et al. (2018) found that females paired with food-deprived males weighed less at the end of a breeding attempt than those paired with well-fed males. This difference is likely a result of the smaller carcass size (8.5–11.5 g) used in Keppner et al. (2018) than in our study (15–20 g), causing greater competition for limited resources among females and males in Keppner et al. (2018). Our results show that females recovered from food deprivation when breeding but that this response was independent of any male assistance. This recovery as well as the lack of an interaction effect may reflect that *N. vespilloides* breeds on small vertebrate carcasses that provide a food source for both parents and offspring (Scott 1998). As such, food-deprived individuals may not require assistance from a partner to recover since they do not have to engage in costly foraging for food from the surrounding environment.

We suggested that whether a species is a capital breeder or an income breeder may be important when considering the effects of nutritional state on parental cooperation. As argued above for our study species, there may be no interaction effects of the focal individual’s nutritional state and that of its partner in capital breeders that acquire resources before breeding. In contrast, we might expect such an interaction effect in income breeders, where parents obtain food to provision to their offspring from the surrounding environment. Previous studies that investigated the effect of food availability on parental behavior in such species, including stitchbirds (Low et al. 2012) and Palestine sunbirds (Markman et al. 2002), have shown differences in parental provisioning rates dependent on manipulation of food availability. One avenue for expanding research in such species is to use two-by-two factorial designs where food availability is manipulated for females and males and then measuring the subsequent effect on parental cooperation. We encourage future work exploring the effect of state on parental cooperation in both capital breeders and income breeders.

Finally, we found no negative effect of parental food deprivation on offspring performance. Instead, average larval mass at dispersal was higher in broods cared for by food-deprived females, and there was no difference in the proportion of larvae surviving to dispersal in response to parental nutritional state. This finding contrasts with that of Keppner et al. (2018), who found no difference in average larval mass dependent on parental nutritional state. Our finding was unexpected given that we predicted that food-deprived parents would provide less care and feed more on the carcass, which is the sole food source for parents and larvae, leading to reduced larval performance upon dispersal. In this species, larval mass at dispersal is a strong predictor of adult size (Lock et al. 2004), which is an important determinant of adult fitness (Otronen 1988). As a result, parents are likely under selection to compensate for any initial reduction of care as a result of poor nutritional state by increased levels of care later in the breeding attempt, or the larvae may compensate by increased rates of self-feeding later in the breeding attempt. Our results suggest overcompensation with food-deprived females producing better quality broods than well-fed females. Our experimental design may have facilitated this since we used relatively large mouse carcasses to ensure that there was ample food for both parents and larvae to feed from without much competition. Thus, there might have been a different outcome, similar to that reported by Keppner et al. (2018), had we used a small carcass such that there was a more intense competition over the shared resource.

In conclusion, our study shows that females respond to both their own and their partner’s nutritional state when deciding how much care to contribute and that these decisions are independent of those made based on the contribution of their partner. In contrast, males responded only to the contribution of their partner rather than the nutritional state of their partner. Our findings highlight the need for more work investigating how multiple mechanisms play a role in the resolution of sexual conflict over parental care and what may be driving sex differences in these mechanisms.

## Data Availability

Analyses reported in this article can be reproduced using the data provided by Lambert and Smiseth (2024).

## References

[CIT0001] Beani L , Bagnères AG, Elia M, Petrocelli I, Cappa F, Lorenzi MC. 2019. Cuticular hydrocarbons as cues of sex and health condition in *Polistes dominula* wasps. Insectes Soc. 66:543–553.

[CIT0002] Brooks ME , Kristensen K, van Benthem KJ, Magnusson A, Berg CW, Nielsen A, Skaug HJ, Mächler M, Bolker BM. 2017. glmmTMB balances speed and flexibility among packages for zero-inflated generalized linear mixed modeling. The R Journal. 9:378–400.

[CIT0003] Eggert AK , Reinking M, Müller JK. 1998. Parental care improves offspring survival and growth in burying beetles. Anim Behav. 55:97–107. 9480676 10.1006/anbe.1997.0588

[CIT0004] Fedina TY , Kuo TH, Dreisewerd K, Dierick HA, Yew JY, Pletcher SD. 2012. Dietary effects on cuticular hydrocarbons and sexual attractiveness in drosophila. PLoS One. 7:e49799. 23227150 10.1371/journal.pone.0049799 PMC3515564

[CIT0005] Fox J , Weisberg S. 2019. An {R} companion to applied regression. Thousand Oaks (CA): Sage.

[CIT0006] Harrison F , Barta Z, Cuthill I, Székely T. 2009. How is sexual conflict over parental care resolved? A meta-analysis. J Evol Biol. 22:1800–1812. 19583699 10.1111/j.1420-9101.2009.01792.x

[CIT0007] Harrison XA. 2015. A comparison of observation-level random effect and Beta-Binomial models for modelling overdispersion in Binomial data in ecology & evolution. PeerJ. 3:e1114. 26244118 10.7717/peerj.1114 PMC4517959

[CIT0008] Hinde CA. 2006. Negotiation over offspring care?—a positive response to partner-provisioning rate in great tits. Behav Ecol. 17:6–12.

[CIT0009] Houston AI , Davies NB. 1985. The evolution of cooperation and life-history in the dunnock. In: Sibly RM, Smith RH, editors. Behavioural Ecology. Oxford: Blackwell. p. 471–87.

[CIT0010] Houston AI , Székely T, McNamara JM. 2005. Conflict between parents over care. Trends Ecol Evol. 20:33–38. 16701338 10.1016/j.tree.2004.10.008

[CIT0011] Johnstone RA , Hinde CA. 2006. Negotiation over offspring care—how should parents respond to each other’s efforts? Behav Ecol. 17:818–827.

[CIT0012] Keppner EM , Ayasse M, Steiger S. 2018. Manipulations of parental nutritional condition reveals competition among family members. J Evol Biol. 31:822–832. 29573021 10.1111/jeb.13266

[CIT0013] Keppner EM , Prang M, Engel KC, Ayasse M, Stökl J, Steiger S. 2017. Beyond cuticular hydrocarbons: chemically mediated mate recognition in the subsocial burying beetle *Nicrophorus vespilloides*. J Chem Ecol. 43:84–93. 28028746 10.1007/s10886-016-0806-8

[CIT0014] Lambert GA , SmisethPT. 2024. Flexible females: nutritional state influences biparental cooperation in a burying beetle. Behav Ecol. doi:10.5061/dryad.s7h44j1f238456179

[CIT0015] Lendvai AZ , Akçay C, Stanback M, Haussmann MF, Moore IT, Bonier F. 2018. Male parental investment reflects the level of partner contributions and brood value in tree swallows. Behav Ecol Sociobiol. 72:185.

[CIT0016] Lessells CM. 2012. Sexual conflict. In: Royle NJ, Smiseth PT, Kölliker M, editors. The evolution of parental care. Oxford: Oxford University Press. p. 119–132.

[CIT0017] Lock JE , Smiseth PT, Moore AJ. 2004. Selection, inheritance, and the evolution of parent-offspring interactions. Am Nat. 164:13–24. 15266367 10.1086/421444

[CIT0018] Low M , Makan T, Castro I. 2012. Food availability and offspring demand influence sex-specific patterns and repeatability of parental provisioning. Behav Ecol. 23:25–34.

[CIT0019] Markman S , Pinshow B, Wright J. 2002. The manipulation of food resources reveals sex-specific trade-offs between parental self-feeding and offspring care. Proc Biol Sci. 269:1931–1938. 12350256 10.1098/rspb.2002.2118 PMC1691109

[CIT0020] Mattey SN , Smiseth PT. 2015. Complex effects of inbreeding on biparental cooperation. Am Nat. 185:1–12. 25560549 10.1086/679067

[CIT0021] McNamara JM , Gasson CE, Houston AI. 1999. Incorporating rules for responding into evolutionary games. Nature. 401:368–371. 10517633 10.1038/43869

[CIT0022] McNamara JM , Wolf M. 2022. Social interaction can select for reduced ability. Proc Biol Sci. 289:20221788. 36259207 10.1098/rspb.2022.1788 PMC9579777

[CIT0023] Müller JK , Eggert AK. 1990. Time-dependent shifts between infanticidal and parental behavior in female burying beetles: a mechanism of indirect mother-offspring recognition. Sociobiology. 27:11–16.

[CIT0024] Otronen M. 1988. The effect of body size on the outcome of fights in burying beetles (*Nicrophorus*). Ann. Zool. Fenn. 25:191–201.

[CIT0025] Parker GA. 1985. Models of parent-offspring conflict. V. Effects of the behaviour of the two parents. Anim Behav. 33:519–533.

[CIT0026] Pilakouta N , Richardson J, Smiseth PT. 2015. State-dependent cooperation in burying beetles: parents adjust their contribution towards care based on both their own and their partner’s size. J Evol Biol. 28:1965–1974. 26245748 10.1111/jeb.12712

[CIT0027] R Core Team. 2021. R: a language and environment for statistical computing. Vienna (Austria): R Foundation for Statistical Computing.

[CIT0028] Ratz T , Smiseth PT. 2018. Flexible parents: joint effects of handicapping and brood size manipulation on female parental care in *Nicrophorus vespilloides*. J Evol Biol. 31:646–656. 29468774 10.1111/jeb.13254

[CIT0029] Richardson J , Smiseth PT. 2019a. Effects of variation in resource acquisition during different stages of the life cycle on life-history traits and trade-offs in a burying beetle. J Evol Biol. 32:19–30. 30311711 10.1111/jeb.13388 PMC7379983

[CIT0030] Richardson J , Smiseth PT. 2019b. Nutrition during sexual maturation and at the time of mating affects mating behaviour in both sexes of a burying beetle. Anim Behav. 151:77–85.

[CIT0031] Schwagmeyer PL , Mock DW, Parker GA. 2002. Biparental care in house sparrows: negotiation or sealed bid? Behav Ecol. 13:713–721.

[CIT0032] Scott MP. 1998. The ecology and behavior of burying beetles. Annu Rev Entomol. 43:595–618. 15012399 10.1146/annurev.ento.43.1.595

[CIT0033] Shippi AG , Paquet M, Smiseth PT. 2018. Sex differences in parental defence against conspecific intruders in the burying beetle *Nicrophorus vespilloides*. Anim Behav. 136:21–29.

[CIT0034] Smiseth PT. 2017. Parental care. In: Reference module in life sciences. Elsevier. Available from: https://www.sciencedirect.com/science/article/pii/B9780128096338124045.

[CIT0035] Smiseth PT. 2019. Coordination, cooperation, and conflict between caring parents in burying beetles. Front Ecol Evol. 7.

[CIT0036] Smiseth PT , Darwell CT, Moore AJ. 2003. Partial begging: an empirical model for the early evolution of offspring signalling. Proc Biol Sci. 270:1773–1777. 12964978 10.1098/rspb.2003.2444 PMC1691438

[CIT0037] Smiseth PT , Dawson C, Varley E, Moore AJ. 2005. How do caring parents respond to mate loss? Differential response by males and females. Anim Behav. 69:551–559.

[CIT0038] Smiseth PT , Moore AJ. 2002. Does resource availability affect offspring begging and parental provisioning in a partially begging species? Anim Behav. 63:577–585.

[CIT0039] Smiseth PT , Moore AJ. 2004. Behavioral dynamics between caring males and females in a beetle with facultative biparental care. Behav Ecol. 15:621–628.

[CIT0040] Steiger S , Peschke K, Francke W, Müller JK. 2007. The smell of parents: breeding status influences cuticular hydrocarbon pattern in the burying beetle *Nicrophorus vespilloides*. Proc Biol Sci. 274:2211–2220. 17609182 10.1098/rspb.2007.0656 PMC2706201

[CIT0041] Venables WN , Ripley BD. 2022. Modern applied statistics with s. New York: Springer.

[CIT0042] Walling CA , Stamper CE, Smiseth PT, Moore AJ. 2008. The quantitative genetics of sex differences in parenting. Proc Natl Acad Sci USA. 105:18430–18435. 19008350 10.1073/pnas.0803146105 PMC2587554

[CIT0043] Ward RJS , Cotter SC, Kilner RM. 2009. Current brood size and residual reproductive value predict offspring desertion in the burying beetle *Nicrophorus vespilloides*. Behav Ecol. 20:1274–1281.

